# The impact of agarose immobilization on the activity of lytic *Pseudomonas aeruginosa* phages combined with chemicals

**DOI:** 10.1007/s00253-022-12349-4

**Published:** 2023-01-10

**Authors:** Agata Dorotkiewicz-Jach, Paweł Markwitz, Jarosław Rachuna, Michał Arabski, Zuzanna Drulis-Kawa

**Affiliations:** 1 Department of Pathogen Biology and Immunology, University of Wroclaw, Przybyszewskiego 63/77, 51-148, Wroclaw, Poland; 2 Institute of Biology, Jan Kochanowski University, Uniwersytecka 7, 25-406, Kielce, Poland

**Keywords:** Agarose immobilization, Phages, Cupric ions, Gentamicin, HDMF furanone, *Pseudomonas aeruginosa*

## Abstract

**Abstract:**

The implementation of non-traditional antibacterials is currently one of the most intensively explored areas of modern medical and biological sciences. One of the most promising alternative strategies to combat bacterial infections is the application of lytic phages combined with established and new antibacterials. The presented study investigates the potential of agarose-based biocomposites containing lytic Pseudomonas phages (KT28, KTN4, and LUZ19), cupric ions (Cu^2+^), strawberry furanone (HDMF), and gentamicin (GE) as antibacterials and anti-virulent compounds for novel wound dressings. Phages (KT28, KTN4, LUZ19, and triple-phage cocktail) alone and in combination with a triple-chemical mixture (Cu + GE + HDMF) when applied as the liquid formulation caused a significant bacterial count reduction and biofilm production inhibition of clinical P. aeruginosa strains. The immobilization in the agarose scaffold significantly impaired the bioavailability and diffusion of phage particles, depending on virion morphology and targeted receptor specificity. The antibacterial potential of chemicals was also reduced by the agarose scaffold. Moreover, the Cu + GE + HDMF mixture impaired the lytic activity of phages depending on viral particles’ susceptibility to cupric ion toxicity. Therefore, three administration types were tested and the optimal turned out to be the one separating antibacterials both physically and temporally. Taken together, the additive effect of phages combined with chemicals makes biocomposite a good solution for designing new wound dressings. Nevertheless, the phage utilization should involve an application of aqueous cocktails directly onto the wound, followed by chemicals immobilized in hydrogel dressings which allow for taking advantage of the antibacterial and anti-virulent effects of all components.

**Key points:**

*• The immobilization in the agarose impairs the bioavailability of phage particles and the Cu + GE + HDMF mixture.*

*• The cupric ions are toxic to phages and are sequestrated on phage particles and agarose matrix.*

*• The elaborated TIME-SHIFT administration effectively separates antibacterials both physically and temporally.*

**Supplementary Information:**

The online version contains supplementary material available at 10.1007/s00253-022-12349-4.

## Introduction

Antibiotics remains the standard treatment option against bacterial infections for decades, but the overuse of those drugs in medicine, agriculture, and the food industry has led to the emergence of multidrug resistance (MDR) among the most life-threatening bacterial pathogens (Aslam et al. 2018). In this light, *Pseudomonas aeruginosa* deserves special consideration as an opportunistic pathogen with high adaptability to hostile environments and intrinsic and acquired resistance mechanisms against common antibiotics (Pang et al. 2019). *P. aeruginosa* causes serious infections, especially in immunocompromized and cystic fibrosis (CF) patients (Church et al. 2006; Breidenstein et al. 2011). During infection, the pathogen employs two strategies: (i) the planktonic cells are highly invasive and induce an acute inflammatory response, whereas (ii) biofilm-forming consortia cause recurrent chronic infections (Valentini et al. 2018). The biofilm structure protects bacteria from harsh environmental conditions including chemicals, antibiotics, and immune system components making the infection difficult to eradicate (Lebeaux et al. 2014; Moradali et al. 2017).

Non-traditional antibacterial therapies are currently intensively explored for utilization in modern medicine. Nowadays, several alternative methods including quorum sensing (QS) inhibitors, lectin inhibitors, metal chelators, nanoparticles, or vaccine designs are already available in clinics or being investigated (Fernebro 2011; Dorotkiewicz-Jach et al. 2015). One of the most promising approaches to combat bacterial infections is the application of lytic phages (bacterial viruses) that infect and replicate within bacterial cells leading to bacterial lysis upon release of progeny phage. Phages do not target eukaryotic cells, and can therefore be applied safely in humans for bacterial infection treatment. They are also intensively explored in the newly emerging field of bio-nanomedicine since the viral particles create precisely defined composition and surface properties (Farr et al. 2014; Paczesny and Bielec 2020). In addition, contrary to antibiotics, bacterial viruses are self-propagating particles at the site of infection and can be even more effective against sessile bacteria if equipped with specific depolymerases that degrade the biofilm matrix (Chatterjee et al. 2016). Phages are already successfully administrated intravenously, by local injection, orally, inhaled, or topically as creams, liquid, or dressing formulations against the plethora of pathogens (Zhvania et al. 2017; Pires et al. 2020). Moreover, many commercial phage products are currently marketed for the clinical, veterinarian, and environmental applications (Duplessis and Biswas 2020). Although modern phage therapies offer the opportunity to provide targeted antibacterial agents with higher specificity than most antibiotics, phage treatment is also restricted by specific limitations. The most often discussed cons of phage therapy include (I) the requirement for pre-diagnosis to allow a rational and personalized therapy, (II) neutralization by the mononuclear phagocyte system and the development of neutralizing antibodies, or (III) inaccessibility of phage to target intracellular bacteria (Melo et al. 2020). Nevertheless, phage therapies are widely investigated due to their therapeutic potential as cocktails with an extended-spectrum activity or combined with other agents to obtain a synergistic antibacterial effect (Knezevic and Sabo 2019).

Taking into consideration all the benefits of phage therapies and the growing problem of *P. aeruginosa* multidrug-resistant biofilm-associated infections, we have decided to investigate the potential use of agarose-based biocomposites combining phages with different antibacterial and anti-virulence compounds. In this regard, topical administration is currently widely investigated as a convenient treatment option for chronically infected wounds, including venous stasis, burn-mediated, and diabetic ulcers. Hydrogels, as carriers for different antibacterials against wound infections, can be composed of different compounds. Agarose is the most commonly used substance in the production of hydrogel dressings as it is characterized by properties similar to native articular cartilage through chondrogenesis and an increased accumulation of glycosaminoglycans. The fibrous structure of agarose allows it to be used as a scaffold for repair and tissue regeneration. Hydrogel dressings constructed from agarose are used as carriers for various substances such as nanoparticles, antibiotics, or bacteriophages (Yang et al. 2018). Agarose is a highly bioactive natural marine polysaccharide with reversible thermogelling behavior, desirable mechanical properties, and switchable chemical reactivity for functionalization (Khodadadi Yazdi et al. 2020).

In our previous study, we investigated liquid combinations of cupric ions (Cu^**2+**^), strawberry furanone (4-hydroxy-2,5-dimethyl-3(2H)-furanone; HDMF), gentamicin (GE), and the three lytic phages (myovirus KT28, giant virus KTN4, podovirus LUZ19) against planktonic *P. aeruginosa* PAO1 strain (Dorotkiewicz-Jach et al. 2021). Cupric ions and gentamicin served as known antimicrobials against *P. aeruginosa* (Hobman and Crossman 2015; Vincent et al. 2016, 2018; Pietsch et al. 2020), whereas the strawberry furanone was used to interfere with two *P. aeruginosa* QS systems (Las and Rhl) and to reduce biofilm formation and other virulence determinants’ production (Choi et al. 2014; Dorotkiewicz-Jach et al. 2015). Our previous study revealed that the best additive anti-pseudomonal effect was obtained for the Cu + GE mixture, due to the efficient binding of cupric ions to the amino groups of aminoglycosides and the enhanced disruption of the bacterial outer membrane or simultaneous activity of both antibacterials elevating the ROS level in the cell (Suwalsky et al. 1998; Kozłowski et al. 2005; Poole 2017). Moreover, the Cu + GE composition and associated oxidation activity might be applied for topical infections, for instance, as a component of modern wound dressings to combat biofilm-related infections (Ahire et al. 2016; Ashfaq et al. 2017; Li et al. 2019). Conversely, to the results published by other groups (Choi et al. 2014), our study confirmed an anti-virulence activity of HDMF but only in the stress-dependent response manner, when bacterial treatment with the Cu and GE imposed oxidative and selective stress respectively, forcing cells to activate protection mechanisms such as copper export, ion sequestration by different molecules, and oxidase production (Kozłowski et al. 2005; Hobman and Crossman 2015; Ladomersky and Petris 2015; Quintana et al. 2017; Novoa-Aponte et al. 2019; Giachino and Waldron 2020). Our previous study showed that the combination of Cu + HDMF gave a significant reduction in the virulence level, especially for QS-regulated elastase and pyocyanin (Dorotkiewicz-Jach et al. 2021). Although this combination revealed a limited antibacterial effect, the reduction of virulence determinants production by a single cell unit was exceptional, confirming the anti-virulence activity of HDMF when specifically combined with stressor agents (Dorotkiewicz-Jach et al. 2021).

The other aspect of our experiments aimed to investigate the impact of tested chemicals on the lytic activity of *P. aeruginosa* phages to verify the potential therapeutic combination in in vitro model. Previously, it was shown that the combination of sub-MIC concentration of GE inhibited an efficient phage progeny production although the antibiotic itself did not neutralize phages. Therefore, to obtain effective phages-gentamycin treatment, both antibacterials should be administrated sequentially. In addition, a phage-drug combination is also considered beneficial with the assumption that the phage-resistant clones remain sensitive to antibiotic action. On the other hand, cupric ions turned out to impair viral particle infectivity in a phage-dependent manner, due to a Cu^**2+**^-DNA intercalation mechanism as reported previously for other viruses (Sagripanti et al. 1991; Reina et al. 2020).

For the present study, the antibacterial and anti-biofilm properties of the triple-chemical mixture (5 mM Cu^**2+**^ + 1 µg/ml GE + 10 µM HDMF) in combination with lytic phages (KT28, KTN4, LUZ19, and triple-phage cocktail) were tested against a panel of *P. aeruginosa* clinical strains. In addition, we investigated whether immobilization in agarose gel as a wound dressing scaffold would impact the activity of the antimicrobials tested.

## Materials and methods

### Bacterial strain and phages

*P. aeruginosa* PAO1 (ATCC 15692) and ATCC 27853 strain from the American Type Culture Collection; clinical strains PAK, 15108/-1, AA43, A5803, 39016, 13121/-1, and CHA; and environmental strains Prr335 and Jpn1563 from international reference panel of *P. aeruginosa* isolates were used in this study (Table 1) (De Soyza et al. 2013; Cullen et al. 2015). Bacteria were stored at − 70 °C in Trypticase Soy Broth (TSB, Becton Dickinson and Company, Cockeysville, MD, USA) supplemented with 20% glycerol. Isolation, propagation, and purification of phages were described elsewhere (Danis-Wlodarczyk et al. 2015). The phage titer was assessed using the double-agar layer technique and purified samples were stored at 4 °C. Phage characteristics are presented in Table 2. Distribution of phages for research purposes is possible upon request to the Department of Pathogen Biology and Immunology, University of Wroclaw, Wroclaw, Poland (KT28 and KTN4 phages) and the Laboratory of Gene Technology, KU Leuven, Leuven, Belgium (LUZ19 phage).

**Table 1:** *P. aeruginosa* strains characteristics

*P. aeruginosa* strains	Source/type of infection	Phage typing (Cullen et al. 2015)		
		KT28*	KTN4*	LUZ19**
PAO1 (ATCC 15692)	Reference strain, wound isolate	+ / −	+ / −	+ / −
ATCC 27853	Reference strain, widely used for antibiotics susceptibility testing	NT	NT	NT
PAK	Clinical isolate from non-CF infection	+ / −	+ / −	+ / −
15108/-1	Clinical isolate from acute infection (ICU)	−	+ / −	+ / −
AA43	Clinical isolate from late CF infection	+ / −	+	−
A5803	Clinical isolate from community-acquired pneumonia	−	+	+ / −
Prr 335	Isolate from the hospital environment	−	+ / −	+ / −
39016	Clinical isolate from a severe keratitis eye infection	−	−	−
Jpn 1563	Environmental isolate from Lake Tamaco	−	+ / −	+ / −
13121/-1	Clinical isolate from acute infection (ICU)	−	−	−
CHA	Clinical isolate from CF	+ / −	+ / −	+ / −

*CF*, cystic fibrosis; *ICU*, intensive care unit, + confluent clear lysis; + / − confluent opaque lysis; − no activity; *NT*, not tested; *collection of the Department of Pathogen Biology and Immunology, University of Wroclaw, Wroclaw, Poland; **collection of the Laboratory of Gene Technology, KU Leuven, Leuven, Belgium

**Table 2:**
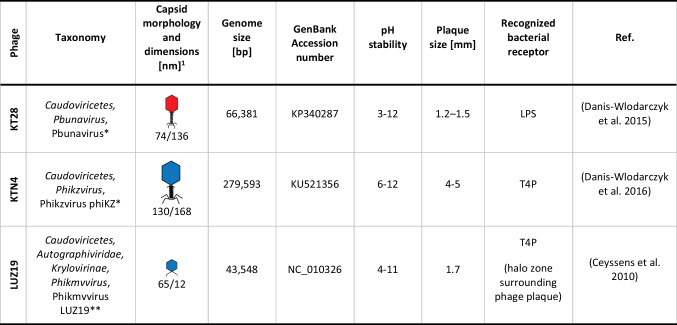
Characteristics of phages selected for this study

^1^Approximated dimensions of head diameter/tail length; *collection of the Department of Pathogen Biology and Immunology, University of Wroclaw, Wroclaw, Poland; **collection of the Laboratory of Gene Technology, KU Leuven, Leuven, Belgium

### Phage lytic activity against *P. aeruginosa* strains

The lytic activity of KT28, KTN4, and LUZ19 phages and triple-phage cocktail (1:1:1; titer:titer:titer) were examined against all selected *P. aeruginosa* strains. For phage experiments, TSB-refreshed bacterial cultures were used. To determine bacterial susceptibility to phage-mediated lysis, a 10-µl aliquot of the phage suspension (10^4^, 10^5^, 10^6^, 10^7^, and 10^8^ PFU/ml, corresponding to 10^2^, 10^3^, 10^4^, 10^5^, and 10^6^ PFU/10 µl drop, respectively) was spotted on a TSA medium using the double-agar layer method and incubated at 37 °C. Plates were examined after 18 h for the presence of a lysis zone (Danis-Wlodarczyk et al. 2015).

### Growth of *P. aeruginosa* strains in the presence of phages, Cu^2+^, gentamycin, and HDMF in combination

According to previously made observations (Dorotkiewicz-Jach et al. 2021), cupric ions at 5 mM, gentamicin at 1 µg/ml, and strawberry furanone at 10 µM concentrations were chosen for experiments. Copper(II) nitrate hemi(pentahydrate) (Cu(NO_3_)_2_ × 2.5 H_2_O) (Cu), 4-hydroxy-2,5-dimethyl-3(2H)-furanone (HDMF), and gentamicin (GE) were obtained from Sigma-Aldrich Chemie GmbH (Steinheim, Germany). The growth of *P. aeruginosa* strains was estimated by measuring the optical density (OD_600_) as described previously (Dorotkiewicz-Jach et al. 2021). Namely, tested combinations (Cu 5 mM + GE 1 µg/ml + HDMF 10 µM; later abbreviated as Cu + GE + HDMF) were prepared in a TSB medium. A 1000 µl solution of Cu + GE + HDMF was transferred to the 24-well titration plate. The 18-h culture on Trypticase Soy Agar (TSA, Oxoid, Basingstoke, UK) was suspended in saline at an optical density of 0.5 McF (~ 5 × 10^8^ CFU/ml) and diluted in a Cu + GE + HDMF sample to establish the starting CFU/ml = 10^6^. Bacterial cultures were incubated alone or inoculated with single phages KT28, KTN4, and LUZ19 or a triple-phage cocktail at the multiplicity of infection 10 (MOI = 10). The growth was monitored in a microplate reader (Varioscan LUX, Thermo Fisher Scientific, Waltham, USA) while incubated for 20 h at 37 °C with agitation. The absorbance measurement (*λ* = 600 nm) was performed automatically every 20 min. Each assay was repeated in triplicate. The results were displayed as cumulative OD_600_ of 20-h growth.

### The inhibitory effect of phages and Cu + GE + HDMF in combination on *P. aeruginosa* biofilm production

To test anti-biofilm activity, a compound combination (Cu + GE + HDMF) was prepared in a TSB medium and 1 ml of the sample was transferred to the 24-well titration plate. A starting CFU/ml = 10^6^ of each culture was prepared as described in the “Growth of *P. aeruginosa* strains in the presence of phages, Cu^2+^, gentamycin, and HDMF in combination” section. The bacterial culture was infected with phages KT28, KTN4, and LUZ19 or triple-phage cocktail at MOI = 10 and incubated for 20 h at 37 °C with agitation. To evaluate the biofilm biomass quantification, plates were washed with saline solution and biofilm was stained with 0.1% crystal violet (CV) for 20 min, washed with distilled water, and the dye was extracted from the biofilm using 96% ethanol solution. The absorbance of CV was measured at 595 nm using a microplate reader (Varioscan LUX, Thermo Fisher Scientific, Waltham, USA)(O’Toole 2011).

### Measurement of phage particles release from agarose using cultivation and laser interferometry methods

To prepare the agarose-immobilized phages in 24-well plates, the 0.7% low melting point agarose (Sigma-Aldrich, St. Louis, MO, USA) in the distilled water was mixed with KT28, KTN4, or LUZ19 phages to a final 10^8^ or 10^9^ PFU/ml at 39 °C. The release of phages from solidified agarose was measured by biological (cultivation) and physical (laser interferometry) methods (Danis-Wlodarczyk et al. 2015; Quintana-Sanchez et al. 2022).

For the biological method, 1 ml of saline was added to 24-well plates containing agarose-immobilized phages. Plates were incubated with agitation at 37 °C for 20 h. The titer of the phages released from agarose to saline was monitored at times 0, 3, and 20 h of incubation, using the spot method on a TSA medium with the double-agar layer technique as described previously (Dorotkiewicz-Jach et al. 2021). Each assay was repeated in triplicate.

Physical laser interferometry was used in parallel. The interferometry system consisted of a two-beam Mach–Zehnder interferometer with a He–Ne laser type HN 40P (Zeiss, Oberkochen, Germany), a cuvette (internal dimensions, 70 mm high, 10 mm wide; optical path length, 7 mm) made with optical glass of high uniformity, a TV-CCD camera, and a computer with software for the acquisition and processing of interference images (interferograms) (Arabski et al. 2007; Olszak et al. 2018; Rewak‐Soroczynska et al. 2021). The agarose-immobilized phages were placed at the bottom of the cuvette which was then filled with water (initial substance concentration *C*_*0*_ = 0). The transport properties of phages from an agarose gel to water were measured based on obtained interferograms and a computer image-processing system. Moreover, taking a series of pictures of interference images over time and subsequent mathematical analysis enables quantitative analysis of real-time release kinetics and concentration distribution of phages in near-agarose gel surface fields. The interferograms, which appear due to the interference of laser beams, were determined by the refraction coefficient of the solute which in turn depends on substance concentration. A concentration gradient generates a distribution of the refractive index in the solution and causes the interference fringes to be bent. The software examines the course of interference fringes, determines the deviation from their straight-line run, and calculates the values of the refractive index at different points in the solution. Using the relation between the substance concentration and the refractive index (determined through a refractometer in a separate experiment), the space–time distribution of concentration is determined. Since the concentration *C(x,t)* and the refraction coefficient are assumed to be linear (Dworecki et al. 2006), the equation is:

1 $$\mathrm C\left(\mathrm x,\mathrm t\right)={\mathrm C}_0+a\triangle n={\mathrm C}_0+\mathrm a\frac{\lambda d\left(\mathrm x,\mathrm t\right)}{\mathrm{hf}},$$

where *C(x,t)* denotes the phage concentration at a point situated at the distance *x* from the agarose gel-water interface; *C*_*0*_ is the initial phage concentration (*C*_*0*_ = 0); *a* is the proportionality constant between the concentration and the refraction index (*a* = 1.6 × 10^14^ 1/l for the phage KT28, *a* = 1.4 × 10^15^ 1/l for the phage KTN4, and *a* = 1.4 × 10^16^ 1/l for the phage LUZ19 aqueous solution); *λ* is the wavelength of the laser light 632.8 nm; *h* is the distance between the fringes in the field where they are straight lines; and *f* is the thickness of the solution in the measurement cuvette. By recording the interferograms over a given time interval, one can reconstruct the concentration profiles at different times. The interferograms were recorded from 120 to 4800 s with a time interval of *∆t* = 120 s and the concentration profiles for each interferogram were reconstructed. Based on concentration profiles, one can determine the transport parameters of substance quantity, such as substance quantity after time *t* (*N(t)*):

2 $$\mathrm N\left(\mathrm t\right)=\mathrm S\int_0^{\mathrm l}\mathrm C\left(\mathrm x,\mathrm t\right)\mathrm{dx},$$

where *S* is the surface of the agarose gel-water interface (S = 7 × 10^−5^ m^2^), and *l* is the height of the measurement cuvette. All experiments were performed at 37 °C.

### The anti-biofilm activity of phages released from agarose on mature *P. aeruginosa* PAO1 biofilm

The mature *P. aeruginosa* PAO1 biofilm was formed in six-well plates (Multiwell Cell Culture Plate, Becton Dickinson, NJ, USA) for 24 h at 37 °C in a TSB medium. Following the incubation, planktonic cells were removed by washing with saline using a shaker at 100 rpm for 10 min. Polyethylene terephthalate (PET) membrane with a pore diameter of 1 µm (as an element of the Cell Culture Insert, Becton Dickinson, NJ, USA) containing the agarose-immobilized phages was placed in the culture well and wells were supplemented with 3 ml of fresh TSB medium. Eradication of bacterial cells and biofilm was monitored for the next 24 h. The *P. aeruginosa* PAO1 cells in the biofilm were stained with Filmtracer™ LIVE/DEAD™ Biofilm Viability Kit (Life Technologies, Carlsbad, CA, USA) according to manufacturer’s instruction and visualized using a fluorescence microscope (Zeiss Axio Scope.A1, Carl Zeiss Canada Ltd. Toronto, Canada). Each assay was repeated at least in triplicate. The representative image of biofilm structure with the highest similarity (the level of aggregation with the distribution and proportion of green (alive) and dead (red) bacterial cells) from 4 images in each repeat (3) was presented.

### The effect of various administrations on antibacterial activity of free or agarose-immobilized phages combined with Cu + GE + HDMF against *P. aeruginosa*

**Fig. 1:**
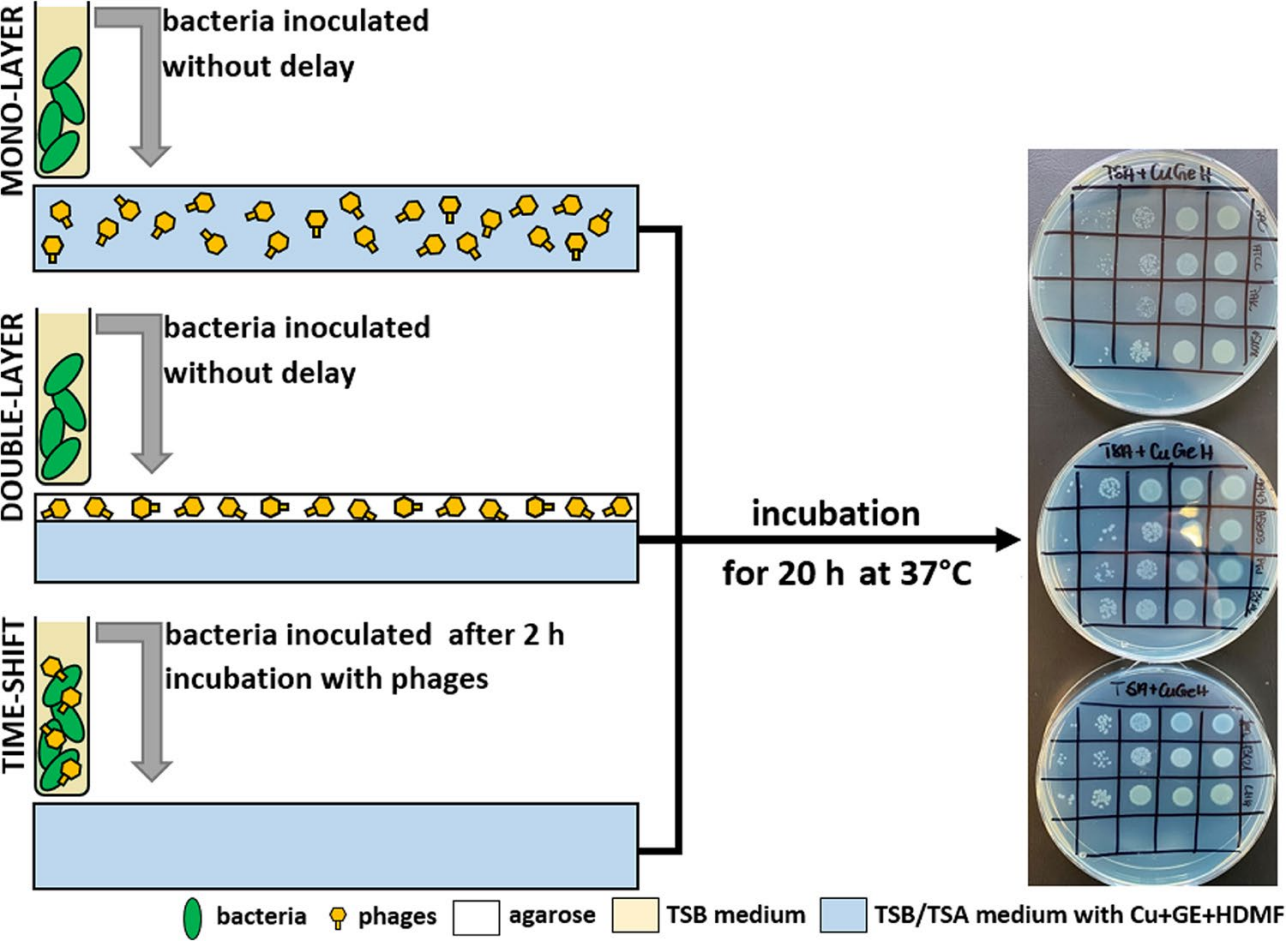
Scheme of phage various administrations in the presence of Cu + GE + HDMF in agarose medium to test the activity against *P. aeruginosa*

Three different types of administration (mono-layer, double-layer, time-shift) of phages (alone and as a triple-phage cocktail) combined with Cu + GE + HDMF were investigated. The scheme of the experiment is presented in Fig. 1.

The 18-h culture of *P. aeruginosa* strains on TSA was suspended in saline to an optical density of 0.5 McF (~ 5 × 10^8^ CFU/ml) and serial dilution was prepared (corresponding to 10^1^,10^2^, 10^3^, 10^4^, and 10^5^ CFU/10 µl drop). For the MONO-LAYER procedure, plates with 20 ml of TSA medium (1.5% of agarose) mixed with Cu + GE + HDMF with or without phages KT28, KTN4, and LUZ19 or triple-phage cocktail were prepared to a final titer of 10^7^ PFU/ml in the medium. Chemicals and phages were added to the liquid medium at the temperature of 39 °C and poured on Petri dishes. Right after, 10 µl of bacterial dilutions was spotted on the plate. Cultures were incubated for 20 h at 37 °C and examined for the CFU/ml reduction compared to the untreated negative control (TSA medium without compounds nor phages).

For the DOUBLE-LAYER procedure, plates with 20 ml of TSA medium (1.5% agarose) supplemented with the stock solution of chemical components at a final concentration of Cu 5 mM, GE 1 µg/ml, and HDMF 10 µM, without phages, were prepared. Chemicals were added to a liquid medium at the temperature of 39 °C and poured on Petri dishes. A separate layer containing phages was prepared next. Phages were suspended in 0.7% low melting point agarose (39 °C) (Sigma-Aldrich, St. Louis, MO, USA) to a final titer of 10^7^ PFU/ml. Next, 5 ml of agarose with phages or phages cocktail was immediately poured on previously prepared plates containing chemical components. Plates without phages were also prepared with a 5-ml layer of 0.7% agarose. Right after, 10 µl of bacterial dilutions was spotted on the plate. Samples were incubated for 20 h at 37 °C and examined for the CFU/ml reduction compared to the untreated negative control (TSA medium without compounds nor phages).

For the TIME-SHIFT procedure, plates with 20 ml of TSA medium supplemented with the stock solution of chemical components at a final concentration of Cu 5 mM, GE 1 µg/ml, and HDMF 10 µM, without phages, were prepared. Chemicals were added to a liquid medium at the temperature of 39 °C and poured on Petri dishes. The 18-h culture of *P. aeruginosa* strains on TSA was suspended in saline to an optical density of 0.5 McF (~ 5 × 10^8^ CFU/ml) and infected with a single phage or triple-phage cocktail at MOI = 10. Bacteria with or without phages were incubated at 37 °C with agitation for 2 h and serial dilutions were prepared (corresponding to 10^1^, 10^2^, 10^3^, 10^4^, and 10^5^ CFU/10 µl drop according to starting CFU/ml). Right after, 10 µl of bacterial dilutions was spotted on the plate. Cultures were incubated for 20 h at 37 °C and examined for the CFU/ml reduction compared to the untreated negative control (TSA medium without compounds nor phages). Each assay was repeated in triplicate.

### Statistical analysis

The data was statistically analyzed using one-way ANOVA and the Levene test, followed by the Tukey test for the experiments measuring the inhibitory effect of phages and Cu + GE + HDMF in combination on *P. aeruginosa* growth and biofilm production. All of the calculations were carried out using the Origin Pro 8.5 software package (OriginLab Corporation). Values of *p* < 0.05 were considered significantly different. Non-parametric statistical analyses such as the Friedman test (non-parametric one-way ANOVA), the Kruskal–Wallis test (one-way ANOVA on ranks), and The Wilcoxon signed-rank test were used to analyze the effect of various administrations on the antibacterial activity of free or agarose-immobilized phages combined with Cu + GE + HDMF*.* Those calculations were carried out using the GraphPad Prism 9.4.1.681 software package (GraphPad Software, San Diego, CA, USA). Values of *p* < 0.05 were considered significantly different.

## Results

**Fig. 2:**
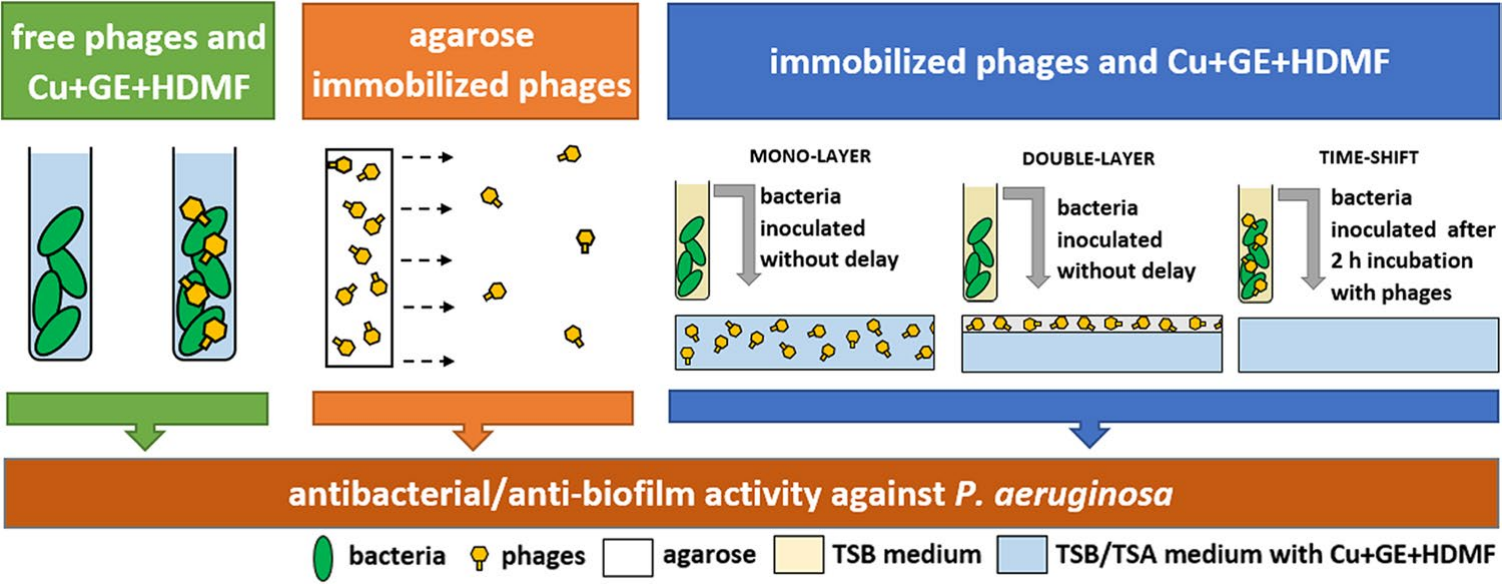
Different aspects of the interactions between the individual components of the designed biocomposite influencing anti-*P. aeruginosa* activity

The presented study aimed to develop a new anti-pseudomonal agarose-based biocomposite combining lytic phages (KT28, KTN4, and LUZ19) with cupric ions, gentamicin, and strawberry furanone (Cu + GE + HDMF), to eradicate or prevent *P. aeruginosa* biofilm formation. The investigation was divided into three stages relating to various aspects of the interactions between the individual components of the designed biocomposite (Fig. 2).

### Phages in a triple cocktail combined with Cu + GE + HDMF mixture are potent anti-pseudomonal agents when applied as free particles

The first stage of the study aimed to determine the antibacterial properties of liquid single phage preparations or triple-phage cocktail separately or in combination with Cu + GE + HDMF against a panel of *P. aeruginosa* clinical strains. In our previous study, a single phage combination with a double-chemical mixture (Cu 5 mM + 1 µg/ml GE; 1 µg/ml GE + 10 µM HDMF; or Cu 5 mM + 10 µM HDMF) showed additive action against reference PAO1 strain at sub-MIC concentrations depending on the phage and composition variants tested (Dorotkiewicz-Jach et al. 2021). Therefore, in the current study, a panel of 11 *P. aeruginosa* strains (Table 1) representing isolates from the environment and different types of infections were tested. Bacterial strains were specifically selected to capture the heterogeneity of *P. aeruginosa*, namely the spectrum of phage susceptibility, bacteria virulence potential, and the source of infection (De Soyza et al. 2013; Cullen et al. 2015).

The host range of KT28, KTN4, and LUZ19 phages as single particles and triple-phage cocktail against the selected *P. aeruginosa* strains is presented in Table 3. Only 15108/-1, AA43, 39016, and Jpn1563 strains remained insensitive to single phage infection, whereas the 13121/-1 strain was the only one resistant to the cocktail. For most of the strains, the titer of 10^4^ PFU/ml was sufficient to cause plaques on the bacterial loan (Table S1), except for ATCC 27853, A5803, and 39016 strains, which required a titer above 10^6^ PFU/ml.

**Table 3:** The activity of KT28, KTN4, and LUZ19 phages as a liquid single preparation or a cocktail against selected *P. aeruginosa* strains

*P. aeruginosa* strains	The lowest phage titer active (PFU/ml)			
	KT28	KTN4	LUZ19	triple-phage cocktail
PAO1	10^4^	10^4^	10^4^	10^4^
ATCC 27853	10^8^*	10^6^	10^7^	10^6^
PAK	10^4^	10^4^	10^4^	10^4^
15108/-1	No activity	No activity	10^4^	10^4^
AA43	10^4^	10^4^	No activity	10^4^
A5803	10^7^	10^4^	10^4^	10^4^
Prr335	10^5^	10^4^	10^4^	10^4^
39016	10^8^*	10^8^*	No activity	10^8^*
Jpn1563	No activity	10^4^	10^4^	10^4^
13121/-1	No activity	No activity	No activity	No activity
CHA	10^4^	10^5^	10^7^	10^4^

^*^Spot lysis of bacterial lawn at the highest phage titer without visible plaques—possible lysis from without

**Fig. 3:**
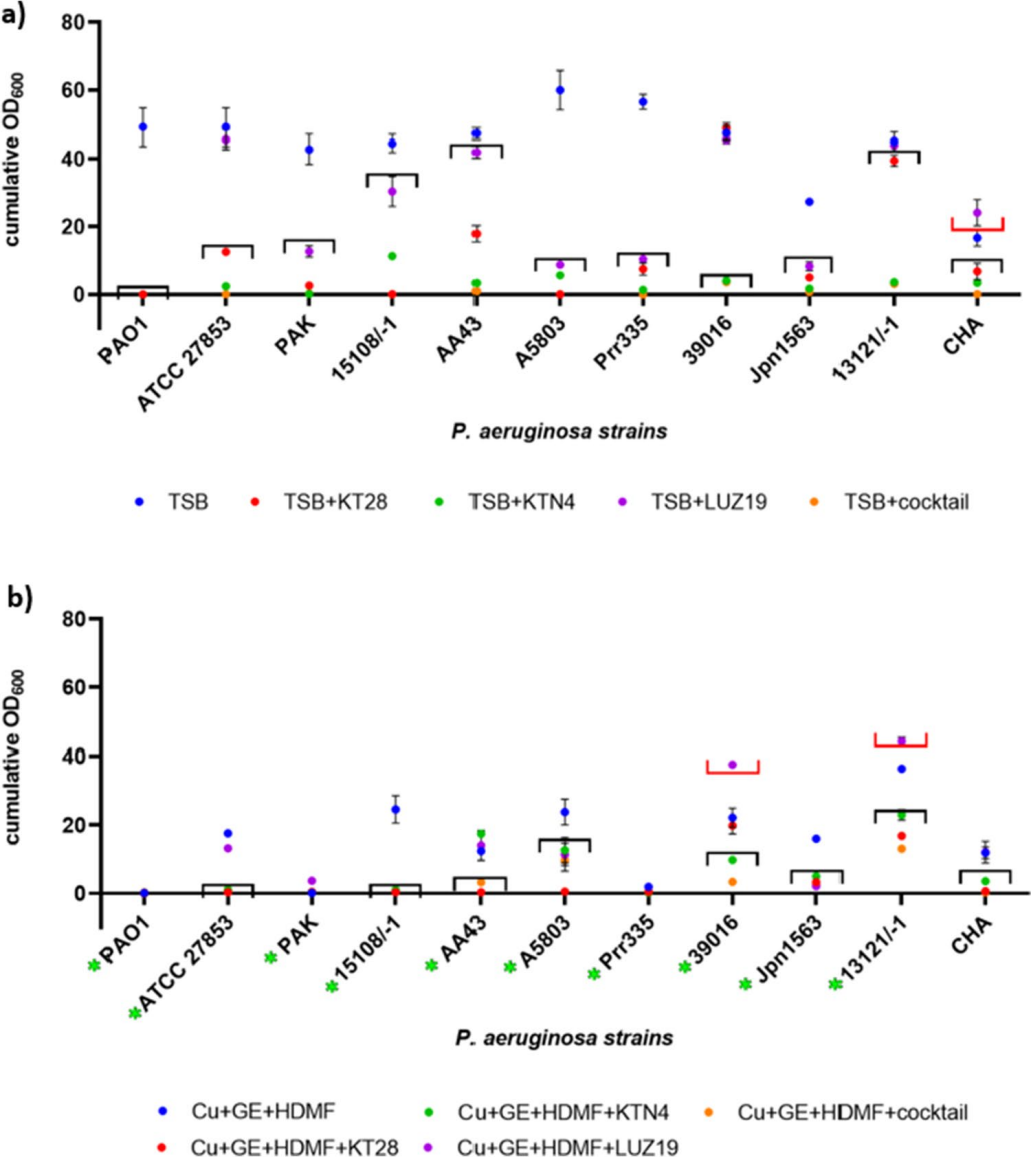
The antibacterial activity of KT28, KTN4, and LUZ19 phages separately, and as a triple-phage cocktail against selected *P. aeruginosa* strains. Results are presented as cumulative OD_600_ after 20 h of incubation in the presence of **a** single/triple-phage cocktail without chemicals, and **b** single/triple-phage cocktail combined with Cu + GE + HDMF. Statistical analyses of tested variants are presented in Supplementary Materials (Table S2). Only the principal statistically significant differences (*p* < 0.05) if compared to **a** TSB or **b** TSB + Cu + GE + HDMF control without phages were presented in the graph as brackets (black represents significantly better antibacterial effect; red represents significantly worse antibacterial effect). The significant antibacterial effect of Cu + GE + HDMF alone against *P. aeruginosa* strains is marked as a green asterisk next to the strain name (panel **b**). The results displayed are the mean of three independent experiments

**Fig. 4:**
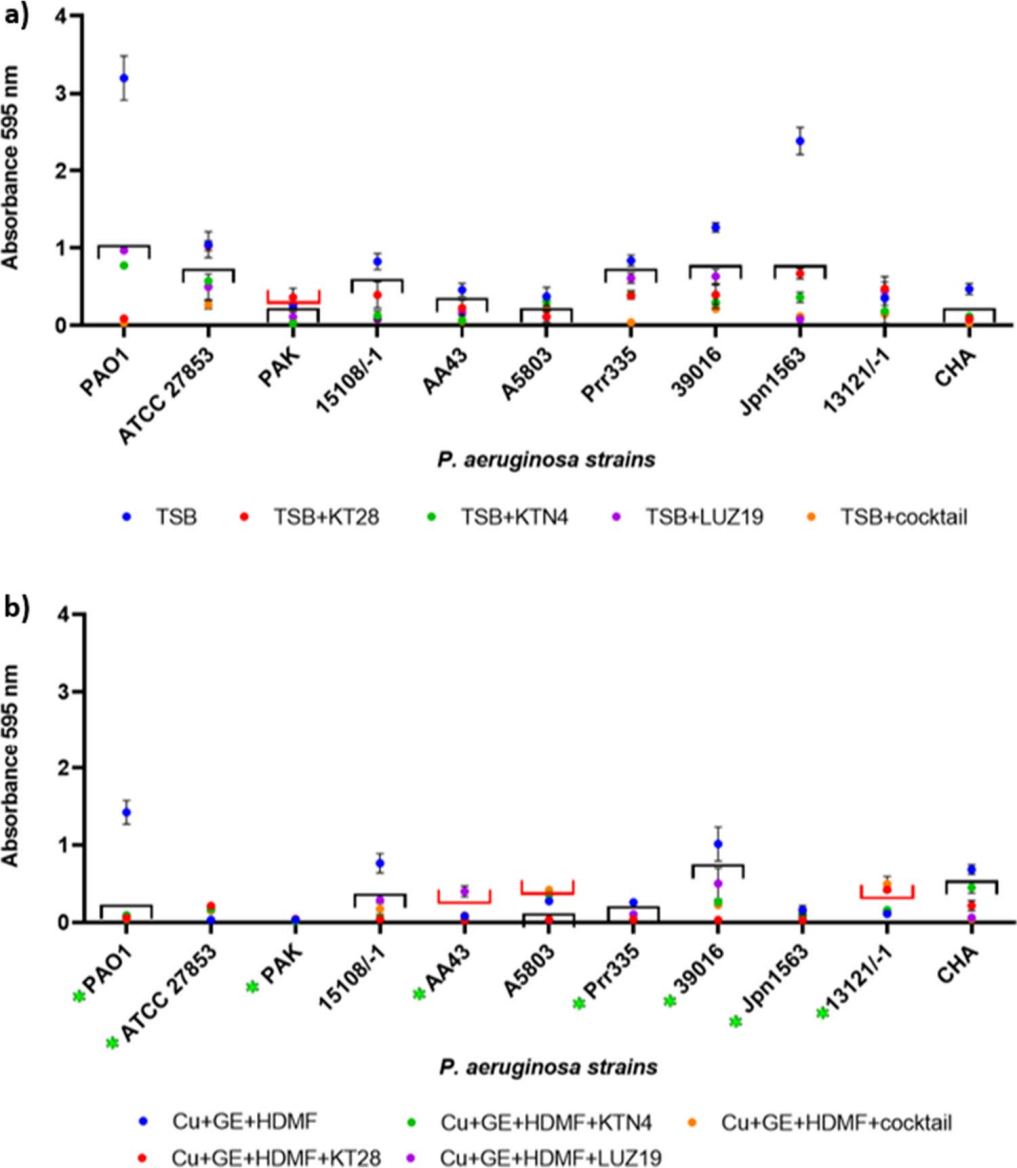
The anti-biofilm activity of KT28, KTN4, and LUZ19 phages separately, and as a triple-phage cocktail against selected *P. aeruginosa* strains. Results are presented as absorbance of CV-stained biofilm biomass after 20 h of incubation in the presence of **a** single/triple-phage cocktail without chemicals, and **b** single/triple-phage cocktail combined with Cu + GE + HDMF. Statistical analyses of tested variants are presented in Supplementary Materials (Table S2). Only the principal statistically significant differences (*p* < 0.05) if compared to **a** TSB or **b** TSB + Cu + GE + HDMF control without phages were presented in the graph as brackets (black represents significantly better anti-biofilm effect, red represents significantly worse anti-biofilm effect). The significant anti-biofilm effect of Cu + GE + HDMF alone against *P. aeruginosa* strains is marked as a green asterisk next to the strain name (panel **b**). The results displayed are the mean of three independent experiments

In the next step, the antibacterial potential of phages combined with Cu + GE + HDMF was determined. Although it was shown in a previous study that cupric ions reduced phage lytic activity in a concentration-dependent manner (Dorotkiewicz-Jach et al. 2021), the goal of this experiment was to test phage combinations with a triple-chemical mixture (at sub-inhibitory concentrations) against the growth and biofilm formation of *P. aeruginosa* clinical strains (Figs. 3 and  4). A statistical analysis of tested variants is presented in Supplementary materials (Table S2 and Table S3). To provide clarity, only statistically significant differences (*p* < 0.05) of tested variants compared to control samples (TSB or TSB + Cu + GE + HDMF) without phages (black and red brackets, respectively) were presented in the graphs since the activity of phages without or with chemicals was the main focus of this study. In addition, significant differences in the activity of TSB + Cu + GE + HDMF compared to control growth in TSB against *P. aeruginosa* strains were marked as a green asterisk (panel “b”). The least effective in reducing the OD_600_ was podovirus LUZ19, which was inactive against strains ATCC 27853, 39016, 13121/-1, and CHA (Fig. 3a). Conversely, the growth of all strains was significantly reduced by the giant KTN4 and the triple-phage cocktail, including the insensitive strain 13121/-1. The freely diffusing phage particles in the broth culture were able to interact with a broader panel of heterogeneous cells in the population, resulting in the overall growth reduction (lowered cumulative OD_600_), although the spot test showed the sensitivity only to a high phage titer or just an insensitivity (Table 3).

The growth of tested *P. aeruginosa* strains, except for the CHA strain, was significantly inhibited in the presence of a chemical mixture (Fig. 3b, marked with a green asterisk next to the strain name) (Table S2, TSB + Cu + GE + HDMF vs TSB variant). These results confirmed the additive effect of all tested chemicals in combination against most of the strains except for A5803 and 13121/-1 (Table S2, triple-phage cocktail + Cu + GE + HDMF vs triple-phage cocktail variant). The significantly reduced antibacterial effect of the single phages in combination with a chemical mixture was observed mainly for KTN4 giant infecting 15108/-1, AA43, 39016, Jpn1563, and 13121/-1 strains. Myovirus KT28 and podovirus LUZ19 combined with Cu + GE + HDMF had a significantly stronger effect on bacterial growth reduction than the individual phages (phage KT28-strains ATCC 27853, AA43, Prr335, 39016, 13121/-1, CHA; phage LUZ19-ATCC 27853, PAK, 15108/-1, AA43, Prr335, 39016, Jpn1563, CHA).

The second aim was to analyze the anti-biofilm activity of the combination variants (Fig. 4). The CV staining of 20-h biofilms of control samples (without phages nor chemicals, Fig. 4a) revealed that 5/11 strains were weak biofilm producers (absorbance 595 nm below 0.5). Significant biofilm inhibition was observed for myovirus KT28 (except ATCC 27853 and PAK strains), giant KTN4, podovirus LUZ19, and triple-phage cocktail applications. Interestingly, phage LUZ19 was able to eradicate the biofilm (ATCC 27853, AA43, 39016, 13121/-1, CHA) even without the culture growth inhibition (lack of the cumulative OD_600_ reduction). This may suggest the activity of probable phage-borne depolymerase (halo zone around phage plaques) causing the degradation of the biofilm matrix. The biofilm production was also significantly prevented by a chemical mixture (Fig. 4b) except for strains 15108/-1, A5803, and CHA (Table S3, TSB + Cu + GE + HDMF vs TSB variant). The addition of the triple-phage cocktail to the chemical mixture further reduced biofilm production for most of the strains excluding A5803 and 13121/-1 where the treatment increased the biofilm biomass (Table S3, cocktail + Cu + GE + HDMF vs cocktail variant, red bracket). A similar situation was observed in some cases (AA43, A5803, and 13121/-1 strains) when single phages (KTN4 or LUZ19) were combined with chemicals (Table S3). These results confirmed our previous observations that cupric ions harmed viral particles’ infectivity in a phage-dependent manner (Dorotkiewicz-Jach et al. 2021). Low susceptibility to Cu^2+^ toxicity was seen for myovirus KT28 whereas giant phage KTN4 and podovirus LUZ19 were more prone to inactivation by cupric ions (Dorotkiewicz-Jach et al. 2021).

### Agarose immobilization reduces the availability of phage particles

The second stage of the study aimed to assess whether hydrogel immobilization as a model material for wound dressing will affect phage bioavailability and lytic potential. We decided to use agarose as a carrier system by the assumption that an electrostatic neutral hydrogel will limit the potential interactions of studied antibacterials with a matrix structure. Moreover, agarose represents outstanding mechanical properties, high bioactivity, and switchable chemical reactivity for functionalization (Khodadadi Yazdi et al. 2020). Nevertheless, its scaffold in a concentration-dependent manner (matrix density) might affect the release of immobilized phage particles. Another issue could be related to possible interactions of agarose polysaccharides with phages recognizing sugar moieties as the receptor. Three different phages (Table 2) were used to ascertain the efficacy of the agarose diffusion rate depending on the phage virion size and virion morphotype (myovirus, giant, podovirus), affinity to polysaccharide versus proteinous receptors (LPS versus T4P), and the sensitivity to cupric ions.

**Fig. 5:**
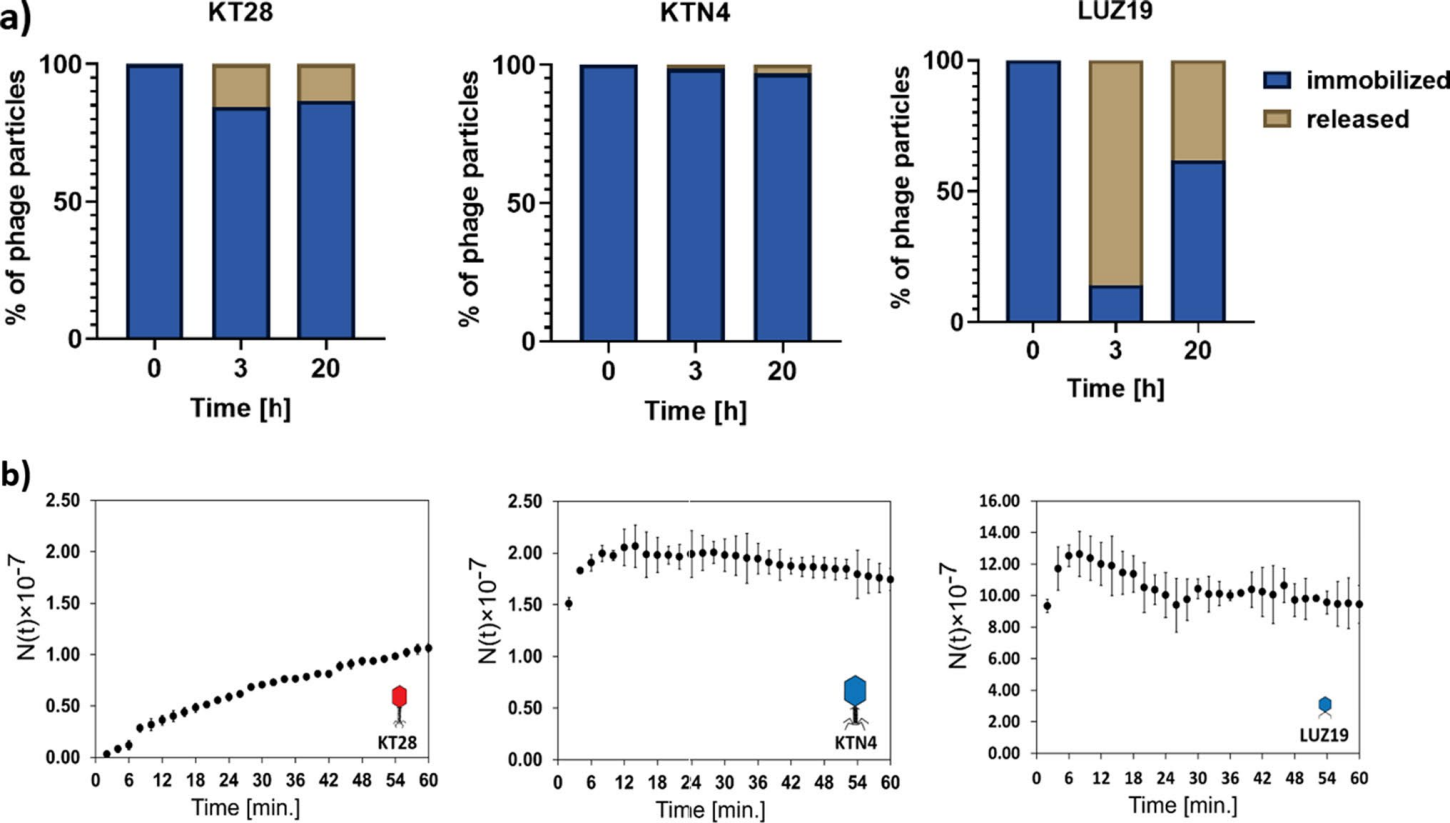
The release of KT28, KTN4, or LUZ19 phages from agarose gel measured by **a** biological method with the PFU/ml determination during 20 h, and **b** physical method (laser interferometry) for 60 min with time interval Δ*t* = 2 min. The results displayed are the mean of three independent experiments

Phage KT28 recognizes sugar moieties of the LPS O-chain, which could cause interference with the polysaccharide network of agarose gel. Such a sugar affinity effect should not be relevant for phages KTN4 and LUZ19 targeting the T4P proteins. To answer the aforementioned questions, two methods of phage release determination were used: biological and physical. The biological method enabled us to determine the exact PFU number of the released phages. Biological measurements were done after 3 and 20 h of immobilized phage incubation in saline with the starting titer of 10^7^ PFU/ml embedded in the agarose gel (0.7%). The physical interferometry method measured the concentration of particles diffusing from the gel phase into the water phase during the first 60 min with starting phage titer of 10^8^ PFU/ml (Fig. 5).

According to the biological measurement, the highest titer of the released particles was obtained for the smallest podovirus LUZ19, having diameters of 65/12 nm (head/tail) and recognizing T4P protein receptors. LUZ19 phage could freely diffuse in the polysaccharide scaffold of the agarose gel as after 3 h, 85.7% of particles were released. Further incubation was accompanied by the diffusion balance and reduced the number of free particles up to 38.1% after 20 h. In the case of giant phage KTN4 and LPS recognizing phage KT28, the final titer of released particles was about tenfold lower (1-log of reduction) compared to the starting titer, giving 3.1% and 13.3% of free phage particles after 20 h of incubation, respectively. Myovirus KT28 with a medium-sized virion (75/136 nm; head/tail) recognizing sugar moieties was gradually released from the agarose scaffold but the results proved that phage diffusion was disturbed by the interactions between virion particles and the polysaccharide scaffold of the agarose gel. The giant phage KTN4 with the big head and the longest tail (130/168 nm head/tail) appeared to be stuck in the agarose scaffold as the diffusion dynamic was slow, releasing 1.4% and 3.1% of phages after 3 and 20 h, respectively.

The interferometry analysis confirmed observations made with the biological method and revealed that the release kinetics of KTN4 and LUZ19 phages from agarose as a function of time were similar, although the number of free particles differed. In the first stage of the diffusion process, a significant increase in free phages was observed, after which the process remained stable. It suggested that KTN4 and LUZ19 phages were transported from the agarose scaffold by diffusion. Conversely, myovirus KT28 was rather gradually released and the kinetic of its transport indicates interaction with agarose scaffold components.

**Fig. 6:**
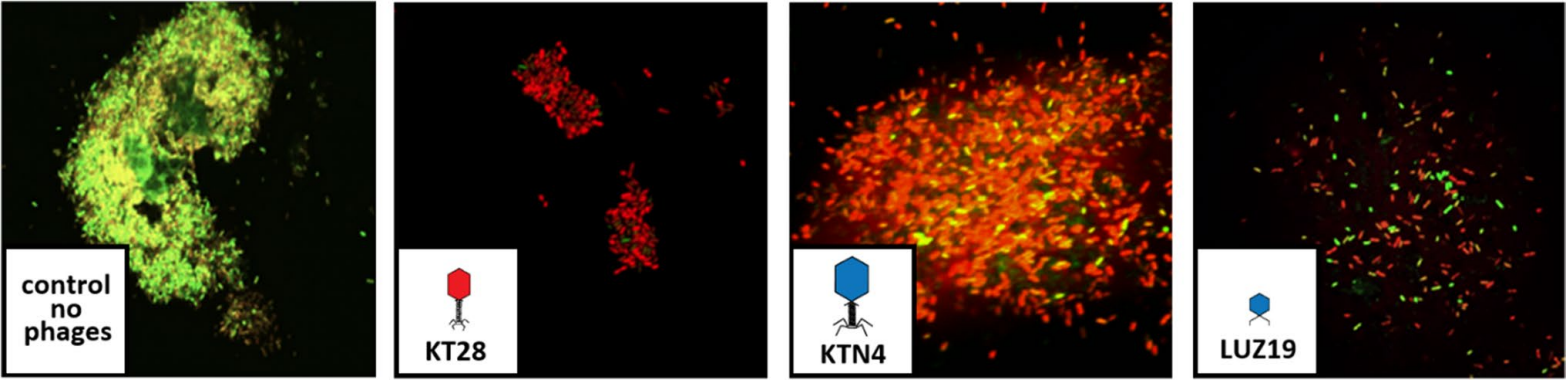
The representative microscopy images of a mature *P. aeruginosa* PAO1 biofilm treated for 24 h with KT28, KTN4, or LUZ19 phages released from agarose gel. Biofilm was stained using Filmtracer™ LIVE/DEAD™ Biofilm Viability Kit, where green bacterial cells are alive, and red cells are dead (magnification 100 ×)

Although KT28 and KTN4 phages displayed reduced release patterns from the agarose gel, the final question was whether the number of available phages is sufficient to obtain effective antibacterial and anti-biofilm activity. The lytic activity of released particles was confirmed by the biological method (plaques enumeration), whereas for anti-biofilm testing, an additional experiment was performed on the mature biofilm of *P. aeruginosa* PAO1 strain as the model organism (Fig. 6). The biofilm treated with podovirus LUZ19 was not as dense as in the case of KTN4 and KT28 phages, which suggested matrix degradation by probable phage-borne depolymerase. A weaker eradication effect for giant phage KTN4 (a portion of cells still alive) was probably a result of a limited phage particle diffusion within the biofilm matrix.

### Triple-phages cocktail administrated with Cu + GE + HDMF mixture immobilized in agarose gel has strong anti-pseudomonal potential

The final aspect of the presented study focused on biocomposite properties, as a model for wound dressing based on an agarose scaffold that combines anti-pseudomonal phages and a Cu + GE + HDMF mixture. Three different ways of compound administration were tested to achieve the optimal additive antimicrobial effect of biocomposite: (1) MONO-LAYER with phages and chemicals combined in the same medium (1.5% of agarose), applied simultaneously; (2) DOUBLE-LAYER with phages immobilized in a separated layer of 0.7% agarose, the components applied simultaneously; (3) TIME-SHIFT with phages applied 2 h ahead and followed with the treatment of immobilized chemicals (Fig. 1). The results of experiments are presented as bacterial count reduction in tenfolds after treatment with a specific administration type (Table 4).

**Table 4:**
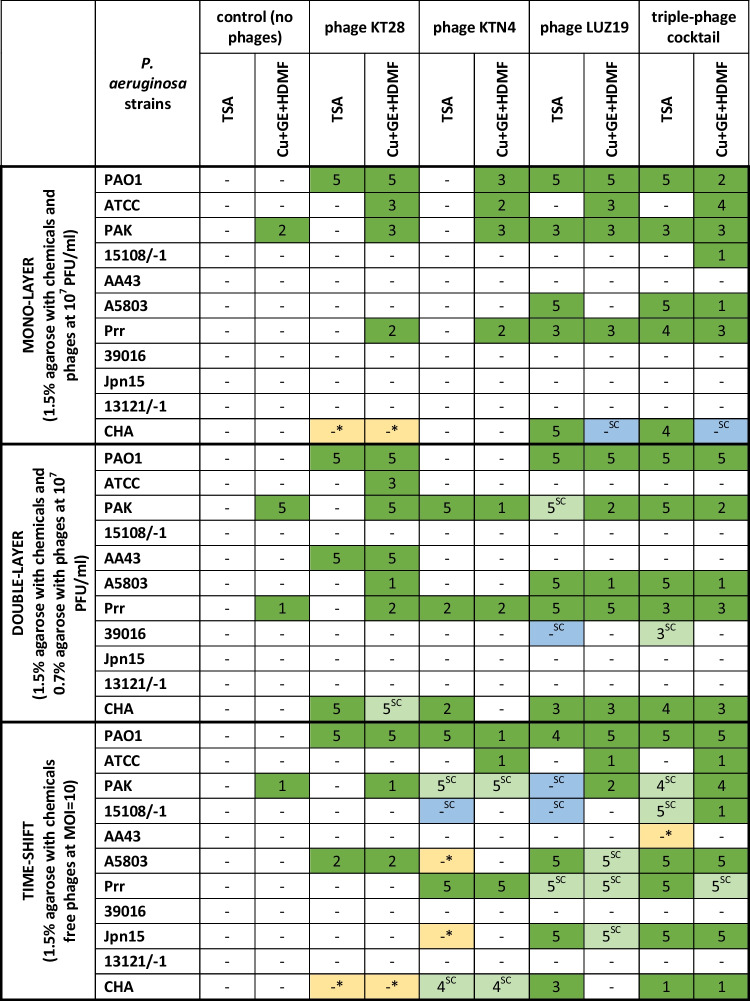
The efficacy of different administrations of single phages, triple-phage cocktail, and a chemical mixture in various combinations against *P. aeruginosa* strains*.* The results are presented as a bacterial count reduction in tenfold (log of reduction). The statistical analyses are presented in Table S5

1–5 (dark green) bacterial count reduction in tenfold; 1–5^SC^ (light green) single clones observed with visible bacterial count reduction in tenfold; -no reduction; -^SC^ (blue) single clones observed with no visible bacterial count reduction in tenfold; *(yellow) no colonies at low culture dilution (1 × and 10 ×) despite the growth at higher dilutions (100 × , 1000 × , 10,000 ×)

Bacterial load was not reduced when chemicals were immobilized in agarose without phages (no significant differences, Table S5), although the growth experiments in the broth culture containing Cu + GE + HDMF showed a significant reduction of the cumulative OD_600_. It may suggest the interactions of the chemical components with the agarose scaffold due to its well-known chemical reactivity. Furthermore, the immobilization of phages reduced their lytic potential due to binding or release blocking from agarose scaffold in a gel concentration-dependent manner. In the MONO-LAYER method (1.5% agarose used for immobilization), phage particle release was insufficient to cause a lytic effect against most of the tested strains and there were no significant differences in bacterial count reduction for phage application versus the control culture growth (Table S5). On the contrary, statistically significant differences were observed in the DOUBLE-LAYER method (0.7% agarose in the upper layer) but only for the triple-phage cocktail, even if a particular strain was sensitive to selected phages. Observed patterns in MONO and DOUBLE-LAYER administrations confirmed that the giant KTN4 did not decrease the bacterial count even for susceptible strains because of the low diffusion rate and limited release due to its virion size. Likewise, phage KT28 did not significantly reduce bacterial load in both types of administrations (with some exceptions) probably due to diffusion limitations related to the interactions with sugar moieties in the agarose scaffold. The smallest, podovirus LUZ19 was the most potent in bacterial count reduction against sensitive strains in both MONO and DOUBLE-LAYER administrations, as their diffusion, within 0.7% agarose gel, was not limited. The triple-phage cocktail provided an additional reduction of the bacterial count compared to single phages, except for the CHA strain. An interesting phenomenon was observed for this strain as no colonies were present at low culture dilutions (1 × and 10 ×), while growth at higher dilutions (100 × , 1000 × , 10,000 ×) was observed.

The significant antibacterial activity of the triple-phage cocktail was achieved in the DOUBLE-LAYER method, apart from the impaired release of all three phages immobilized in one preparation. The TIME-SHIFT method of administration, on the other hand, confirmed that the lack of phage immobilization gave the significantly better activity of the triple-phage cocktail (Table S5) compared to the single particles.

When the Cu + GE + HDMF mixture was combined with phages, various effects were observed in a method-, phage-, and strain-dependent manner. First, we analyzed whether immobilized chemicals (all three administrations) affected phage activity against the entire heterogeneous collection of *P. aeruginosa.* No statistically significant differences were found when phages were applied separately versus in combination with chemicals (Table S5), although the higher bacterial count reduction of individual strains was visible for phage combination with chemicals (Table 4). It was also confirmed that the presence of cupric ions in MONO-LAYER method resulted in the drop of LUZ19 phage and triple-phage cocktail lytic activity when single bacterial strains were compared. The same patterns were seen when phages were separated from the chemicals in the DOUBLE-LAYER administration. Nevertheless, the phage cocktail with Cu + GE + HDMF proved to be the most potent combination in both MONO- and DOUBLE-LAYER administrations although no statistically significant differences were noted.

Second, the difference between the activity of immobilized chemicals versus immobilized chemicals plus phages was analyzed. Several significant differences were observed in the administration method-dependent manner (Table S5). In the MONO-LAYER method, a triple-phage cocktail with a chemical mixture was significantly better (additive effect) than the chemicals alone (Table S5). In the DOUBLE-LAYER, a significantly better lytic activity was observed for slowly releasing phage KT28, which was also resistant to toxic cupric ions at tested concentration. In the TIME-SHIFT method, where phage particles were separated from the chemicals, the most efficient were phage LUZ19 and triple-phage cocktail.

The optimal antibacterial effect was observed for the TIME-SHIFT method, where free phages infected bacteria and propagate during the first 2 h of incubation. The following exposition to chemicals enhanced later the overall antibacterial effect. Physical separation of phages by a TIME-SHIFT of administration from chemicals allowed utilizing of both antibacterial modes of action. On the other hand, phage infection selects bacterial populations for phage-resistant clones, but in our case, only single growing colonies (Table 4, marked as SC) were observed, meaning bacterial load was heavily reduced (Table S4).

Statistical comparison between the antibacterial potential of three different administration methods showed no significant differences (the Friedman and Kruskal–Wallis tests). It means that different conditions employed in tested methods mattered in various ways (agarose density, phage particle size, phage receptor, susceptibility to cupric ion toxicity, lytic phage activity, immobilization, temporal and physical separation).

## Discussion

The presented study was conducted to investigate the properties of an anti-pseudomonal agarose-biocomposite consisting of *Pseudomonas* lytic phages, cupric ions, gentamicin, and strawberry furanone embedded in the agarose scaffold. The designed biocomposite was analyzed for antibacterial and anti-biofilm activity against a relevant panel of *P. aeruginosa* clinical strains. Moreover, the specific interactions between components, meaning phages, chemicals, and the agarose scaffold as an immobilization medium were explored.

First of all, we noticed that the susceptibility of bacterial strains to phage infection varied slightly depending on the method used. The lytic activity of the cocktail without chemical adjuncts was able to successfully target 10 out of 11 strains (excluding 13121/-1 strain) using the spotting method, but these results differed from the broth method (cumulative OD_600_ measurement). In broth culture, all *P. aeruginosa* strains were shown to be sensitive to giant phage KTN4 and triple-phage cocktail infection, which is probably conditioned by free diffusion of large KTN4 particles in the liquid medium. The least effective in broth culture (compared to the agar spot test) was podovirus LUZ19, giving significantly increased cumulative OD_600_ values of spot-sensitive ATCC 27853 and CHA strains. It might be explained by the fast emergence and dispersion of phage-resistant clones that could lack receptors for phage adsorption.

The anti-biofilm activity showed significant inhibition of biofilm production in the presence of myovirus KT28, giant KTN4, podovirus LUZ19, and triple-phage cocktail applications. Interestingly, phage LUZ19 presence inhibited biofilm formation of *P. aeruginosa* ATCC 27853, AA43, 39016, 13121/-1, and CHA strains although they were insensitive to podovirus infection under the tested conditions. That might be also connected to the activity of a putative depolymerase produced by this podovirus. The anti-biofilm effect of LUZ19 particles released from agarose was also confirmed in the biofilm eradication experiment (confocal microscope analysis) where podovirus LUZ19 treatment loosened the biofilm matrix although a portion of bacterial cells was still alive. Our findings are consistent with the results presented by Gula et al. (Guła et al. 2020). Next, we determined the efficacy of chosen chemical compounds in a triple mixture against *P. aeruginosa* strains. The sub-MIC concentrations of GE and Cu^2+^ had significant antibacterial and anti-biofilm activity against most of the strains (10 out of 11) and no adverse interactions between used compounds were observed. Conversely, in our previous study, the HDMF in combination with either Cu^2+^ or GE (chemical stressors) diminished the antibacterial effect but was able to effectively inhibit the production of elastase and pyocyanin by the PAO1 strain (Dorotkiewicz-Jach et al. 2021). The triple-chemical mixture gave both significant bacterial count reduction (measured as cumulative OD_600_) and biofilm production inhibition when broth cultures were tested. The observed effect might be addressed to the discussed phenomenon of additive action of gentamicin and cupric ions (Dorotkiewicz-Jach et al. 2021). We explained that interactions of cupric cations with the amino groups of aminoglycosides gave the enhanced disruption of the bacterial outer membrane. The Cu + GE augmentation with HDMF resulted also in an effective biofilm production inhibition.

The chemical mixture (Cu + GE + HDMF) affected the phages in two opposing ways. The increased antibacterial effect was observed if phages were insensitive to the cupric ion toxicity (myovirus KT28), whereas the bioavailability of sensitive KTN4 and LUZ19 particles was diminished. Consequently, the overall antibacterial potential was reduced in a titer-dependent manner (sequestration of available cupric ions on phage particles) similarly as previously reported (Dorotkiewicz-Jach et al. 2021). Nevertheless, no significantly reduced lytic activity of the phage cocktail combined with chemicals was observed in broth cultures. Moreover, the probable phage-borne depolymerase of phage LUZ19 maintained its anti-biofilm activity in the presence of a chemical mixture. The observed reduced biofilm production might be also additively addressed to the HDMF interference with *P. aeruginosa* quorum sensing (QS) systems. Choi et al*.* previously proved that among four *P. aeruginosa* QS systems, two (Rhl and Las) are under the control of acyl-homoserine lactone (AHL) signals which are competing with HDMF molecules for receptors (Choi et al. 2014).

Since we confirmed the strong antibacterial and anti-biofilm activity of phages in combination with chemicals in liquid cultures, we next addressed the possibility of phage immobilization in an agarose scaffold as the model medium for wound dressing design. It turned out that the agarose scaffold significantly impaired the bioavailability and diffusion of phage particles, depending on virion morphology and recognized receptor. The smallest podovirus LUZ19 was able to freely diffuse from and into agarose gel, maintaining a high titer of released particles. The diffusion of myovirus KT28 recognizing the LPS chains as the receptor was constant in time suggesting the saturation effect, likely due to binding to the sugar scaffold of agarose and the presence of a long tail in the capsid structure. Nevertheless, the phage release was enough efficient to provide antibacterial and anti-biofilm properties. The gel immobilization impaired strongly the availability of free particles of giant KTN4 since around 97% of the whole viral load has been trapped in the gel scaffold due to the phage particle size. We assumed that both morphology/size and recognized receptors influence the potential of immobilized phages. Based on that fact, biocomposite dressing materials should be designed considering the diameter of the applied scaffold and the charge of components. Moreover, the high phage titer should be immobilized to overcome possible diffusion hurdles and to provide the maximum of available free viral particles at the site of infection sufficient to eradicate the pathogen. In the study by Wright et al., the treatment of *P. aeruginosa*-associated chronic otitis a phage cocktail containing six phages at 10^5^ PFU/ml was sufficient to observe clinical improvements relative to the placebo group (Wright et al. 2009). In our study, the final titer of phages released from the agarose scaffold was 1-log lower than the immobilized load, resulting in 10^6^ PFU/ml and 10^7^ PFU/ml for biological and physical determination, respectively.

The last part of this work aimed to investigate the possibility of combining phages with a Cu + GE + HDMF mixture in the agarose scaffold of the designed anti-pseudomonal nano-biocomposite, since agarose is currently widely used in various research fields due to its thermo-reversible gel-forming ability, making it a straightforward model for microbiological applications (Guastaferro et al. 2021). Three administration types were selected (MONO-LAYER, DOUBLE-LAYER, and TIME-SHIFT). Unfortunately, the antibacterial activity of chemicals alone was not observed when dissolved in an agarose scaffold compared to a liquid formulation (no bacterial count reduction). This was probably related to the interactions of cationic chemicals with negatively charged sugar moieties of the agarose scaffold. The diffusion characteristics of various substances from agarose gels (among them antibiotics and metal ions) relate to the rheological properties of the gels which can be easily modified (Kim et al. 2019). In our study, low melting agarose was used to deliver the substance quickly due to the reduced double helicoidal structure of the scaffold and subsequent lower storage modulus (Kim et al. 2019). For future applications, this aspect might be further investigated to design strategies for controlling the release of loaded antibacterials from biomaterial scaffolds.

Phage immobilization mixed with chemicals exhibited similar patterns to those observed in the agarose-releasing model if 0.7% agarose was used. The agarose scaffold limited the diffusion of phage particles within the bacterial population resulting in reduced lytic activity. Although, CFU/ml decrease was observed (*) but not as a typical tenfold reduction, and the emergence of resistant variants was visible. The most effective was podovirus LUZ19, and the least potent was giant KTN4. A thin 0.7% agarose layer in the DOUBLE-LAYER administration improved phage availability compared to the MONO-LAYER version with 1.5% agarose, but reduced diffusion of myovirus and giant affected the overall lytic potential. Due to the impaired release of phage particle from the agarose scaffold, the most efficient administration was first to apply non-immobilized phages followed by agarose-chemicals treatment in the TIME-SHIFT method of administration. The addition of the Cu + GE + HDMF mixture to nano-biocomposite in different types of administration affected the overall antibacterial properties in a phage- and strain-dependent manner. The least effective was the MONO-LAYER method in which phages and chemicals were immobilized together in the 1.5% agarose medium; thus, toxic cupric ions reduced the load of active phages. The DOUBLE-LAYER method separated the immobilized phages from chemical components but both agents were administered at the same time and cupric ions could still affect phages’ infectivity. The physical and temporal separation of antimicrobials in the TIME-SHIFT method allowed for the best effectiveness of components, although chemicals were probably partially neutralized by the agarose scaffold. First, phages were able to infect bacteria without the adverse impact of the cupric ions. Shifted-in-time application of chemicals dissolved in the gel provided an additive antibacterial effect on the bacterial fraction insensitive to phage infection. Our results are consistent with previously discussed studies reporting a beneficial treatment combination of phages and antibiotics (aminoglycoside) but only when components were administrated sequentially (Chaudhry et al. 2017; Torres-Barceló et al. 2018; Dorotkiewicz-Jach et al. 2021). The phage-antibacterial combination is considered beneficial under the assumption that the phage-treated population remains sensitive to drug-resistant bacteria. In addition, as reported by Markwitz et al., increased sensitivity to antibacterials of bacterial populations selected by phage infection might affect the overall activity of the nano-biocomposite administrated in the TIME-SHIFT manner (Markwitz et al. 2021). Moreover, it is worth mentioning that the simultaneous resistance emergence to antibiotics and phages would entail huge metabolic costs, and a slow-growing, less-virulent population would rapidly be cleared by the immune system (Torres-Barceló and Hochberg 2016).

In summary, the presented experiments showed that the immobilization of phages with chemicals in one nano-biocomposite gives an overlapping effect since the cupric ions might lower phage titer, and the agarose scaffold inhibited their release. Therefore, the best solution was to use both physically and temporally separated antibacterials. Moreover, the agarose matrix partially neutralized cupric ions. Nevertheless, applying a high titer of phages for immobilization still provides a significant reduction of bacterial load and biofilm.

All aforementioned aspects of the antibacterial effect of phage combination with chemicals immobilized in the hydrogel would promote the idea of designing phage-based nano-biocomposites for new wound dressings. Nevertheless, the best antibacterial administration was found to be the TIME-SHIFT one where phages are delivered in liquid cocktails followed by chemical antibacterials immobilized in an agarose scaffold. Such a model of TIME-SHIFT administration allows phages to infect bacteria and start propagating; thus, chemicals dissolved in the gel provide an additional antibacterial effect on the bacterial fraction insensitive to phage infection.

## Supplementary Information

Below is the link to the electronic supplementary material.

Supplementary file1 (PDF 899 KB)

## Data Availability

All data generated or analyzed during this study are included in this published article.
