# Supplementary material for: The impact of agarose immobilization on the activity of lytic Pseudomonas aeruginosa phages combined with chemicals
Source: Appl Microbiol Biotechnol. 2023 Jan 10;107(2-3):897–913. doi: 10.1007/s00253-022-12349-4 (PMC9842590; doi:10.1007/s00253-022-12349-4)
Supplement: Supplementary file 1 — Supplementary file1 (PDF 899 KB) [file 253_2022_12349_MOESM1_ESM.pdf]

The impact of agarose immobilization on the activity of lytic *Pseudomonas aeruginosa* phages combined with chemicals

Agata Dorotkiewicz-Jach[1] Pawel Markwitz[1], Jaroslaw Rachuna[2], Michal Arabski[2] and Zuzanna Drulis-Kawa[1*]

Table S1. The activity of KT28, KTN4 and LUZ19 phages as a liquid single preparation or a cocktail against selected *P. aeruginosa* strains

**Phages**		**PFU/10 µl drop**		
		**10^2 10^3 10^4 10^5 10^6**		**10^2 10^3 10^4 10^5 10^6**
**KT28**<br>**KTN4**<br>**LUZ19**<br>**triple-phages cocktail**	***P.aeruginosa* PAO1**	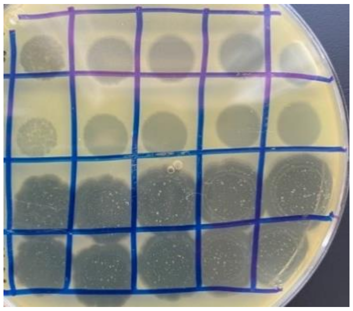	***P.aeruginosa* ATCC 27853**	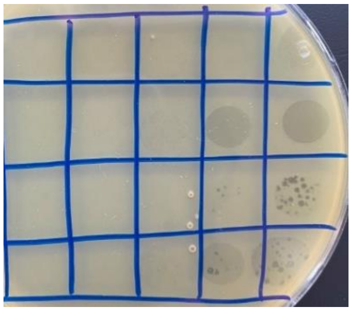
**KT28**<br>**KTN4**<br>**LUZ19**<br>**triple-phages cocktail**	***P.aeruginosa* PAK**	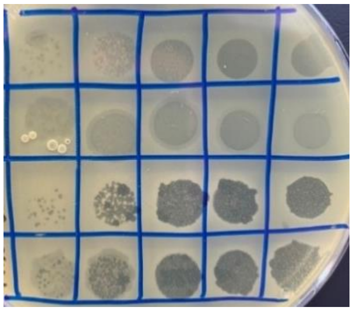	***P.aeruginosa* 15108/-1**	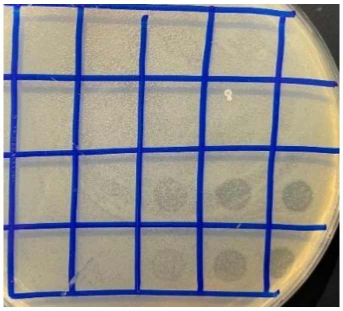
**KT28**<br>**KTN4**<br>**LUZ19**<br>**triple-phages cocktail**	***P.aeruginosa* AA43**	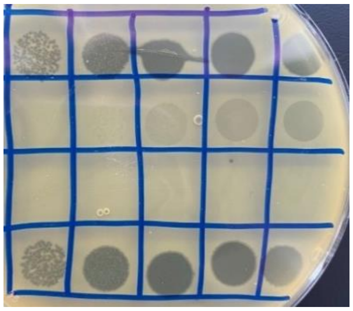	***P.aeruginosa* A5803**	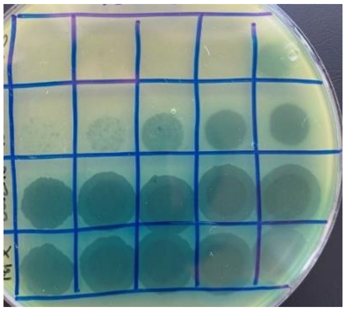

**KT28**<br>**KTN4**<br>**LUZ19**<br>**triple-phages cocktail**	***P.aeruginosa* Prr355**	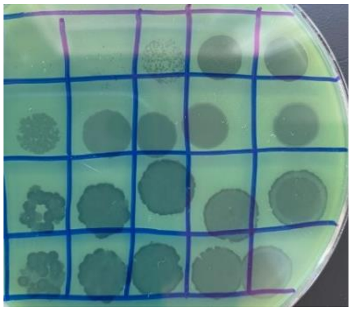	***P.aeruginosa* 39016**	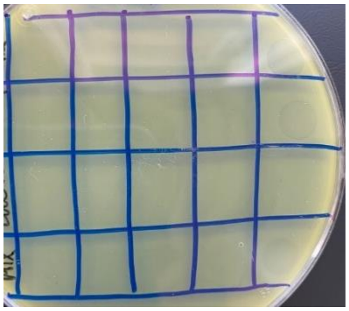
**KT28**<br>**KTN4**<br>**LUZ19**<br>**triple-phages cocktail**	***P.aeruginosa* Jpn1563**	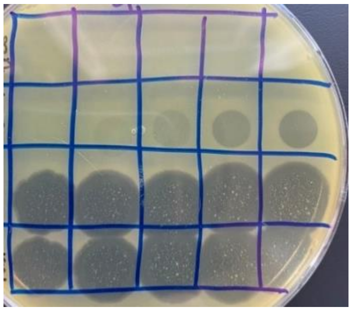	***P.aeruginosa* 13121/-1**	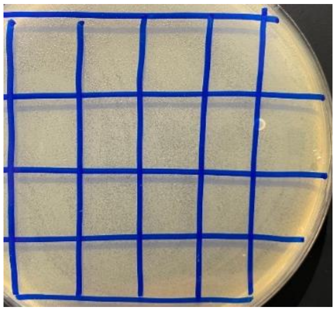
**KT28**<br>**KTN4**<br>**LUZ19**<br>**triple-phages cocktail**	***P.aeruginosa* CHA**	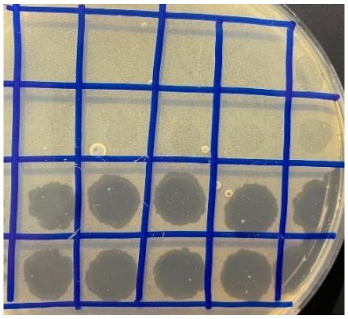		

*lysis of bacterial lawn in spot with highest phage titer without visible plaques

Table S2. Statistical analyses (one-way-ANOVA and the Levene test, followed by the Tukey test) of tested variants for the antibacterial activity of KT28, KTN4 and LUZ19 phages separately, and as a triple-phage cocktail against selected *P. aeruginosa* strains (cumulative OD_{600})

Tested variants	*P. aeruginosa* strains										
	PAO1	ATCC 27853	PAK	15108/-1	AA43	A5803	Prr335	39016	Jpn1563	13121/-1	CHA
**TSB+Cu+GE+HDMF vs TSB**	1	1	1	1	1	1	1	1	1	1	0
**KT28 vs TSB**	1	1	1	1	1	1	1	0	1	1	1
**KT28 vs TSB+Cu+GE+HDMF**	0	0	0	1	1	1	1	1	1	0	0
**KT28+Cu+GE+HDMF vs TSB**	1	1	1	1	1	1	1	1	1	1	1
**KT28+Cu+GE+HDMF vs TSB+Cu+GE+HDMF**	0	1	0	1	1	1	0	0	1	1	1
**KT28+Cu+GE+HDMF vs KT28**	0	1	0	0	1	0	1	1	0	1	1
**KTN4 vs TSB**	1	1	1	1	1	1	1	1	1	1	1
**KTN4 vs TSB+Cu+GE+HDMF**	0	1	0	1	1	1	0	1	1	1	1
**KTN4 vs KT28**	0	1	0	1	1	0	1	1	1	1	0
**KTN4 vs KT28+Cu+GE+HDMF**	0	0	0	1	0	0	0	1	0	1	0
**KTN4+Cu+GE+HDMF vs TSB**	1	1	1	1	1	1	1	1	1	1	1
**KTN4+Cu+GE+HDMF vs TSB+Cu+GE+HDMF**	0	1	0	1	0	1	0	1	1	1	1
**KTN4+Cu+GE+HDMF vs KT28**	0	1	0	0	0	1	1	1	0	1	0
**KTN4+Cu+GE+HDMF vs KT28+Cu+GE+HDMF**	0	0	0	0	1	1	0	1	0	1	0
**KTN4+Cu+GE+HDMF vs KTN4**	0	0	0	1	1	0	0	1	1	1	0
**LUZ19 vs TSB**	1	0	1	1	1	1	1	0	1	0	1
**LUZ19 vs TSB+Cu+GE+HDMF**	0	1	1	0	1	1	1	1	1	1	1
**LUZ19 vs KT28**	0	1	1	1	1	1	1	0	1	1	1
**LUZ19 vs KT28+Cu+GE+HDMF**	0	1	1	1	1	0	1	1	1	1	1
**LUZ19 vs KTN4**	0	1	1	1	1	0	1	1	1	1	1
**LUZ19 vs KTN4+Cu+GE+HDMF**	0	1	1	1	1	0	1	1	1	1	1
**LUZ19+Cu+GE+HDMF TSB**	1	1	1	1	1	1	1	1	1	0	0
**LUZ19+Cu+GE+HDMF vs TSB+Cu+GE+HDMF**	0	0	0	1	0	1	0	1	1	1	0
**LUZ19+Cu+GE+HDMF vs KT28**	0	0	0	0	0	1	1	1	1	1	0
**LUZ19+Cu+GE+HDMF vs KT28+Cu+GE+HDMF**	0	1	0	0	1	1	0	1	0	1	1
**LUZ19+Cu+GE+HDMF vs KTN4**	0	1	0	1	1	0	0	1	0	1	1
**LUZ19+Cu+GE+HDMF vs KTN4+Cu+GE+HDMF**	0	1	0	0	0	0	0	1	1	1	1
**LUZ19+Cu+GE+HDMF vs LUZ19**	0	1	1	1	1	0	1	1	1	0	1

**triple phage cocktail vs TSB**	1	1	1	1	1	1	1	1	1	1	1
**triple phage cocktail vs TSB+Cu+GE+HDMF**	0	1	0	1	1	1	0	1	1	1	1
**triple phage cocktail vs KT28**	0	1	0	0	1	0	1	1	1	1	1
**triple phage cocktail vs KT28+Cu+GE+HDMF**	0	0	0	0	0	0	0	1	1	1	0
**triple phage cocktail vs KTN4**	0	0	0	1	0	0	0	0	0	0	0
**triple phage cocktail vs KTN4+Cu+GE+HDMF**	0	0	0	0	1	1	0	1	1	1	0
**triple phage cocktail vs LUZ19**	0	1	1	1	1	1	1	1	1	1	1
**triple phage cocktail vs LUZ19+Cu+GE+HDMF**	0	1	0	0	1	1	0	1	0	1	1
**triple phage cocktail+Cu+GE+HDMF vs TSB**	1	1	1	1	1	1	1	1	1	1	1
**triple phage cocktail+Cu+GE+HDMF vs TSB+Cu+GE+HDMF**	0	1	0	1	1	1	0	1	1	1	1
**triple phage cocktail+Cu+GE+HDMF vs KT28**	0	1	0	0	1	1	1	1	1	1	1
**triple phage cocktail+Cu+GE+HDMF vs KT28+Cu+GE+HDMF**	0	0	0	0	0	1	0	1	0	1	0
**triple phage cocktail+Cu+GE+HDMF vs KTN4**	0	0	0	1	0	0	0	0	0	1	0
**triple phage cocktail+Cu+GE+HDMF vs KTN4+Cu+GE+HDMF**	0	0	0	0	1	0	0	1	1	1	0
**triple phage cocktail+Cu+GE+HDMF vs LUZ19**	0	1	1	1	1	0	1	1	1	1	1
**triple phage cocktail+Cu+GE+HDMF vs LUZ19+Cu+GE+HDMF**	0	1	0	0	1	0	0	1	0	1	1
**triple phage cocktail+Cu+GE+HDMF vs triple phage cocktail**	0	0	0	0	0	1	0	0	0	1	0

1 significant, 0 not significant

Table S3. Statistical analyses (one-way-ANOVA and the Levene test, followed by the Tukey test) of tested variants for the anti-biofilm activity of KT28, KTN4 and LUZ19 phages separately, and as a triple-phage cocktail against selected *P. aeruginosa* strains (absorbance of CV-stained biofilm biomass)

Tested variants	*P. aeruginosa* strains										
	PAO1	ATCC 27853	PAK	15108/-1	AA43	A5803	Prr335	39016	Jpn1563	13121/-1	CHA
**TSB + Cu+GE+HDMF vs TSB**	1	1	1	0	1	0	1	1	1	1	0
**KT28 vs TSB**	1	0	1	1	1	1	1	1	1	0	1
**KT28 vs TSB + Cu+GE+HDMF**	1	1	1	1	1	1	1	1	1	1	1
**KT28 + Cu+GE+HDMF vs TSB**	1	1	1	1	1	1	1	1	1	0	1
**KT28 + Cu+GE+HDMF vs TSB + Cu+GE+HDMF**	1	0	0	1	0	1	1	1	0	1	1
**KT28 + Cu+GE+HDMF vs KT28**	0	1	1	1	1	0	1	0	1	0	0
**KTN4 vs TSB**	1	1	1	1	1	0	1	1	1	0	1
**KTN4 vs TSB + Cu+GE+HDMF**	0	1	0	1	0	0	1	1	1	0	1
**KTN4 vs KT28**	1	1	1	1	1	1	0	0	1	1	0
**KTN4 vs KT28 + Cu+GE+HDMF**	1	1	0	0	0	1	1	0	1	0	0
**KTN4 + Cu+GE+HDMF vs TSB**	1	1	1	1	1	0	1	1	1	0	0
**KTN4 + Cu+GE+HDMF vs TSB + Cu+GE+HDMF**	1	0	0	1	0	0	1	1	0	0	0
**KTN4 + Cu+GE+HDMF vs KT28**	0	1	1	1	1	1	1	0	1	1	1
**KTN4 + Cu+GE+HDMF vs KT28 + Cu+GE+HDMF**	0	0	0	0	0	1	0	0	0	1	1
**KTN4 + Cu+GE+HDMF vs KTN4**	1	1	0	0	0	0	1	0	1	0	1
**LUZ19 vs TSB**	1	1	1	1	1	1	1	1	1	0	1
**LUZ19 vs TSB + Cu+GE+HDMF**	0	1	0	1	0	1	1	0	0	1	1
**LUZ19 vs KT28**	1	1	1	1	0	0	1	0	1	0	0
**LUZ19 vs KT28 + Cu+GE+HDMF**	1	1	0	0	0	0	1	1	0	0	0
**LUZ19 vs KTN4**	0	0	0	0	0	1	1	0	1	0	0
**LUZ19 vs KTN4 + Cu+GE+HDMF**	1	1	1	0	0	1	1	0	0	1	1
**LUZ19 + Cu+GE+HDMF vs TSB**	1	1	1	1	0	1	1	1	1	0	1
**LUZ19 + Cu+GE+HDMF vs TSB + Cu+GE+HDMF**	1	0	0	1	1	1	1	1	0	0	1
**LUZ19 + Cu+GE+HDMF vs KT28**	0	1	1	0	0	0	1	0	1	1	0
**LUZ19 + Cu+GE+HDMF vs KT28 + Cu+GE+HDMF**	0	0	0	1	1	0	0	1	0	1	0
**LUZ19 + Cu+GE+HDMF vs KTN4**	1	1	0	0	1	1	1	0	1	0	0
**LUZ19 + Cu+GE+HDMF vs KTN4 + Cu+GE+HDMF**	0	0	0	1	1	1	0	0	0	0	1
**LUZ19 + Cu+GE+HDMF vs LUZ19**	1	1	0	0	1	0	1	0	0	1	0

**triple phage cocktail vs TSB**	1	1	1	1	1	0	1	1	1	0	1
**triple phage cocktail vs TSB + Cu+GE+HDMF**	1	1	0	1	0	0	1	1	0	0	1
**triple phage cocktail vs KT28**	0	1	1	1	1	0	1	0	1	1	0
**triple phage cocktail vs KT28 + Cu+GE+HDMF**	0	0	0	0	0	1	0	0	0	0	0
**triple phage cocktail vs KTN4**	1	1	0	0	0	0	1	0	1	0	0
**triple phage cocktail vs KTN4 + Cu+GE+HDMF**	0	0	0	0	0	0	0	0	0	0	1
**triple phage cocktail vs LUZ19**	1	1	0	0	0	0	1	1	0	1	0
**triple phage cocktail vs LUZ19 + Cu+GE+HDMF**	0	0	0	0	1	1	0	0	0	0	0
**triple phage cocktail + Cu+GE+HDMF vs TSB**	1	1	1	1	1	0	1	1	1	0	1
**triple phage cocktail + Cu+GE+HDMF vs TSB + Cu+GE+HDMF**	1	0	0	1	0	1	1	1	0	1	1
**triple phage cocktail + Cu+GE+HDMF vs KT28**	0	1	1	0	1	1	1	0	1	0	0
**triple phage cocktail + Cu+GE+HDMF vs KT28 + Cu+GE+HDMF**	0	0	0	0	0	1	0	0	0	0	0
**triple phage cocktail + Cu+GE+HDMF vs KTN4**	1	1	0	0	0	0	1	0	1	1	0
**triple phage cocktail + Cu+GE+HDMF vs KTN4 + Cu+GE+HDMF**	0	0	0	0	0	0	0	0	0	1	1
**triple phage cocktail + Cu+GE+HDMF vs LUZ19**	1	1	0	0	0	1	1	0	0	0	0
**triple phage cocktail + Cu+GE+HDMF vs LUZ19 + Cu+GE+HDMF**	0	0	0	0	1	1	0	0	0	1	0
**triple phage cocktail + Cu+GE+HDMF vs triple phage cocktail**	0	0	0	0	0	1	0	0	0	1	0

1 significant, 0 not significant

Table S4. The efficacy of different administration of single phages, triple-phage cocktail and a chemical mixture in various combinations against *P. aeruginosa* strains.

	P. aeruginosa **strains**	**no phages (control of growth)**	**immobilized Cu+GE+HDMF mixture**	**immobilized triple-phages cocktail**	**immobilized triple-phages cocktail with Cu+GE+HDMF mixture**
		**inoculated CFU/10 µl drop**			
		**10^1 10^2 10^3 10^4 10^5**	**10^1 10^2 10^3 10^4 10^5**	**10^1 10^2 10^3 10^4 10^5**	**10^1 10^2 10^3 10^4 10^5**
**MONO-LAYER**	**PAO1**<br>**ATCC 27853**<br>**PAK**<br>**15108/-1**<br>**AA43**<br>**A5803**<br>**Prr335**<br>**39016**<br>**Jpn1563**<br>**13121/-1**<br>**CHA**	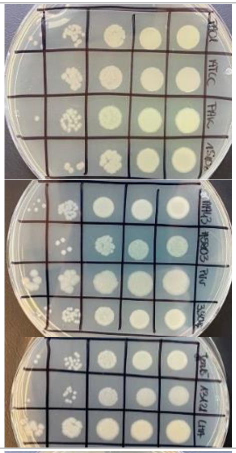	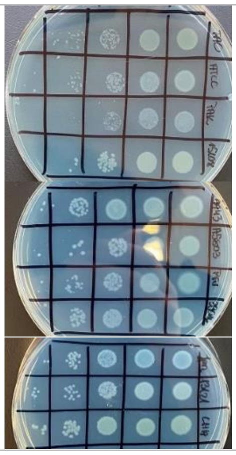	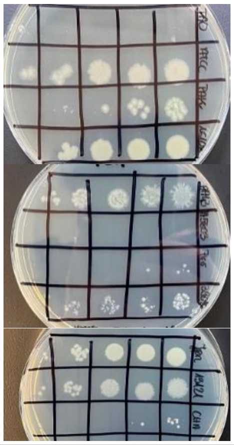	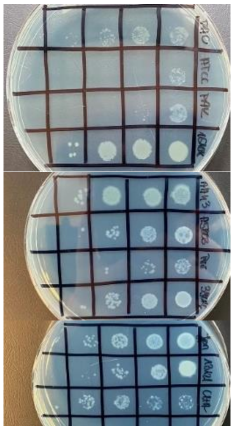
**DOUBLE-LAYER**	**PAO1**<br>**ATCC 27853**<br>**PAK**<br>**15108/-1**<br>**AA43**<br>**A5803**<br>**Prr335**<br>**39016**<br>**Jpn1563**<br>**13121/-1**<br>**CHA**	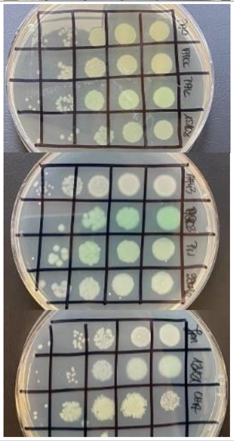	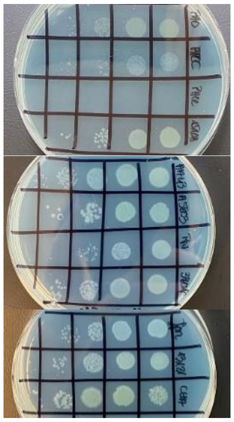	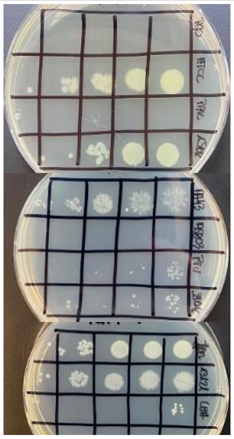	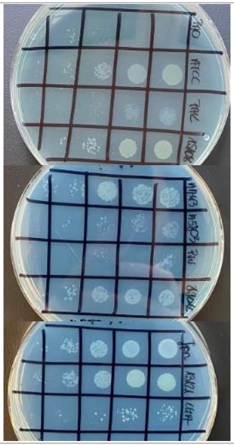
**TIME-SHIFT**	**PAO1**<br>**ATCC 27853**<br>**PAK**<br>**15108/-1**<br>**AA43**<br>**A5803**<br>**Prr335**<br>**39016**<br>**Jpn1563**<br>**13121/-1**<br>**CHA**	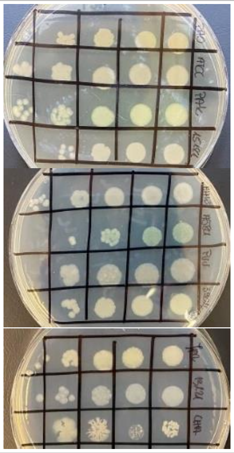	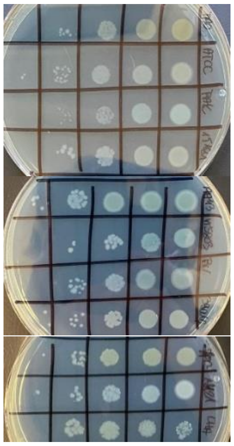	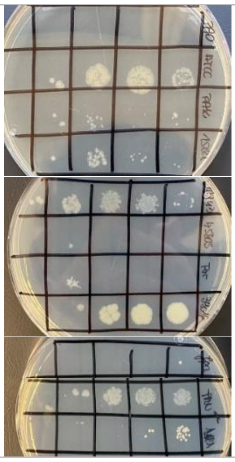	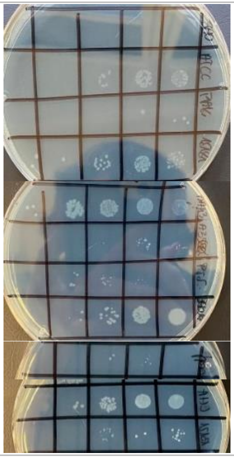

Table S5. The Wilcoxon signed-rank test results used to analyze the effectiveness of different administration methods presented in Table 4.

Tested variants	Method of administration		
	MONO-LAYER	DOUBLE-LAYER	TIME-SHIFT
**TSA_KT28 vs TSA _no_phages**	0	0	0
**TSA _KTN4 vs TSA _no_phages**	0	0	0
**TSA _LUZ19 vs TSA _no_phages**	0	0	0
**TSA _ triple phage cocktail vs TSA _no_phages**	0	1	1
**TSA_Cu+GE+HDMF_no phages vs TSA _no_phages**	0	0	0
**TSA_Cu+GE+HDMF_KTN28 vs TSA _no_phages**	0	0	0
**TSA_Cu+GE+HDMF_KTN4 vs TSA _KTN4**	0	0	0
**TSA_Cu+GE+HDMF_LUZ19 vs TSA _LUZ19**	0	0	0
**TSA_Cu+GE+HDMF_ triple phage cocktail vs TSA _ triple phage cocktail**	0	0	0
**TSA_Cu+GE+HDMF_KTN28 vs TSA_Cu+GE+HDMF _no_phages**	0	1	0
**TSA_Cu+GE+HDMF_KTN4 vs TSA_Cu+GE+HDMF _no_phages**	0	0	0
**TSA_Cu+GE+HDMF_LUZ19 vs TSA_Cu+GE+HDMF _no_phages**	0	0	1
**TSA_Cu+GE+HDMF_ triple phage cocktail vs TSA_Cu+GE+HDMF _no_phages**	1	0	1

1 significant, 0 not significant
